# The Significance of Microenvironmental and Circulating Lactate in Breast Cancer

**DOI:** 10.3390/ijms242015369

**Published:** 2023-10-19

**Authors:** Vincenza Frisardi, Simone Canovi, Salvatore Vaccaro, Raffaele Frazzi

**Affiliations:** 1 Geriatric Unit, Neuromotor Department, Azienda Unità Sanitaria Locale—IRCCS di Reggio Emilia, 42122 Reggio Emilia, Italy; 2 Clinical Laboratory, Azienda Unità Sanitaria Locale—IRCCS di Reggio Emilia, 42122 Reggio Emilia, Italy; 3 Clinical Nutrition Unit and Oncological Metabolic Centre, Azienda Unità Sanitaria Locale—IRCCS di Reggio Emilia, 42122 Reggio Emilia, Italy; 4 Scientific Directorate, Azienda Unità Sanitaria Locale—IRCCS di Reggio Emilia, 42122 Reggio Emilia, Italy

**Keywords:** lactate, aerobic glycolysis, acidosis, breast cancer

## Abstract

Lactate represents the main product of pyruvate reduction catalyzed by the lactic dehydrogenase family of enzymes. Cancer cells utilize great quantities of glucose, shifting toward a glycolytic metabolism. With the contribution of tumor stromal cells and under hypoxic conditions, this leads toward the acidification of the extracellular matrix. The ability to shift between different metabolic pathways is a characteristic of breast cancer cells and is associated with an aggressive phenotype. Furthermore, the preliminary scientific evidence concerning the levels of circulating lactate in breast cancer points toward a correlation between hyperlactacidemia and poor prognosis, even though no clear linkage has been demonstrated. Overall, lactate may represent a promising metabolic target that needs to be investigated in breast cancer.

## 1. Introduction

Lactate gained considerable attention after the discovery that it contributes to oxidative metabolism and functions to satisfy the tumor cell energy demand [1,2]. It is well accepted that cancer cells drive glucose intake and metabolism toward fermentation, gaining fewer ATP molecules but improving the production of metabolic intermediates necessary for anabolic reactions finalized to cell growth [3]. These metabolic processes are driven by the hypoxia-inducible transcription factor HIF or by the oncogenic transcription factor MYC [1]. Consistently, the monocarboxylate transporters (MCTs) catalyze the efflux of lactate (accumulating within cells) contributing to maintaining an intracellular neutral pH while favoring extracellular acidosis [4]. Thus, the MCTs (MCT1 and MCT4 in particular) are strongly associated with the hyperglycolitic phenotype in cancer cells. These transporters are overexpressed/localized in various cancers, including breast cancer, which is the focus of the present manuscript [4]. Accordingly, with the postulated function of lactate, it has been demonstrated that its extracellular concentrations in tumors can be up to 20-fold higher than in non-malignant tissues (about 40 mM vs. 1.5–3 mM, respectively) [5,6,7,8].

The original observation of an increased glucose-dependent production of lactate by tumor cells was made by Otto Warburg one century ago, and a metabolic signature named the “Warburg effect” was established fifty years later [9,10]. For many decades, lactic acid was seen as a mere end product of the glycolytic metabolism of cancer cells. However, in more recent years, it has become evident that lactate metabolism plays a central role in cancer biology. The links between oncogenic molecular tumor pathways (e.g., MYC) and genes regulating lactate metabolism (e.g., LDH) emerged [11], and it has now become clear that lactate plays a role as a metabolic substrate, a signaling molecule, and it has a leading role in a metabolic crosstalk between cancer cells and tumor microenvironment (TME) [12,13]. Overall, lactate represents a perfect example of why the reprogramming of cellular metabolism constitutes a hallmark of cancer biology (Table 1) [14].

The metabolic switch toward aerobic glycolysis (known as the Warburg effect) is also a topical research field in breast cancer (BC) [15]. Triple-negative breast cancers (TNBCs, lacking the expression of estrogen receptor (ER), progesterone receptor (PR), and human epidermal growth factor-2 (HER2)) can benefit from this metabolic switch and, consistently, can be targeted by novel therapeutic compounds called glycolysis inhibitors. Despite this potential therapeutic opportunity, breast tumor cells may evade thanks to the use of alternative metabolic patterns, a phenomenon known as “metabolic plasticity” [15]. The lack of hormone and HER2 targets implies that TNBCs represent the most aggressive subtype and are still treated with chemotherapy-based regimens. The chemoresistance insurgence and related toxicities hamper the efficacy of the treatments, while the hypoxic microenvironment of pre-malignant lesions (as better explained below) leads to a selective pressure toward an aerobic glycolysis switch. In addition to this, the hypoxia-driven changes in the TNBC extracellular matrix positively affect the establishment of the Warburg effect [15].

The current vision supports the functionality of TNBC mitochondria and the ability to switch among different metabolic patterns [16]. Since these same metabolic plasticity phenomena mediate the invasion, proliferation, and metastasis potential of TNBCs, research aimed at targeting lactate as an oncometabolite is of outmost importance. The aim of the present manuscript is to summarize the most relevant roles played by lactate in the microenvironment and in the blood of BC patients.

Studies that provided preclinical evidence regarding microenvironmental lactate or preclinical and clinical evidence regarding circulating lactate in BC patients were included, with a focus on the most relevant and helpful recent studies related to our research topic. We used a narrative approach to describe the relevance of this topic, aiming to spark discussion.

## 2. Lactate and Metabolism in the TME

TME has been characterized over the years as an active player of primary importance for the survival and progression of tumor cells. The modulation of the immune response, cytokine secretion, and angiogenesis are just some of the processes actively modulated through TME [17]. The metabolism of tumor cells is dynamic and influenced by the availability of nutrients and metabolites in the extracellular milieu. Vasculogenesis is crucial for the disease, and, following tumor growth and expansion, some heterogeneous areas arise and metabolic byproducts (lactate and adenosine) accumulate in the TME [17]. The blood and lymphatic vasculature also play a key role regarding immune cells. Vasculature represents physical and functional barriers to the tumor-infiltrating immune cell extravasation (blood vessels) and to the tumor-associated antigen-presenting dendritic cell (DC) drainage (lymphatic vessels) to the lymph nodes [18].

Erratic tumor vasculature is a hallmark of cancer and is the result of angiogenesis [19]. The characteristics of pathological angiogenesis bear a significant contribution to the immunosuppressive TME. The chaotic and leaky organization leads to worse perfusion and nutrient supply, favoring hypoxia and acidification of the tissue [18]. The glycolytic nature of hypoxic tumor cells lowers the pH of the TME in the range of 6.0–6.5, being directly linked to lactate production. It has been demonstrated that this acidosis promotes metastasis, angiogenesis, and immunosuppression, ultimately leading to a worse clinical prognosis [20].

**Figure 1:**
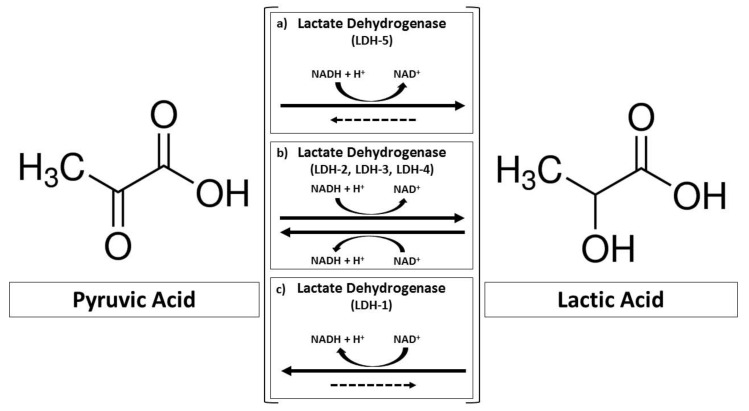
The reversible conversion of pyruvate into lactate catalyzed by LDH. The main isoforms (LDH-1, LDH-2, LDH-3, LDH-4, and LDH-5) are represented.

Metabolism reprogramming is now considered one of the 14 hallmarks of cancer that accompany carcinogenesis [21]. The metabolic phenotype of tumor cells shares features with hypoxia, ischemia, embryonic growth, development, and exercise, among others. This updated view of tumor metabolism also led to a re-evaluation of the role played by lactate produced by tumor cells. Lactate is no longer a byproduct of anaerobic glycolysis but, on the contrary, an active fuel of oxidative metabolism under normal oxygenation conditions (as described below). Lactate produced through aerobic glycolysis was originally believed to serve to regenerate the rate-limiting coenzyme NAD^+^ from NADH for successive rounds of glycolysis by the cytosolic LDH reaction (Figure 1) [1]. The pioneering work by Otto Warburg was further improved and exploited, leading to the discovery that cancer cells, even in the absence of oxygen, produce about two/three of the total ATPs normally obtained during oxidative metabolism by normal cells [22]. In tumor cells, both anaerobic and aerobic glucose metabolism contribute to satisfying the energy demand, implying that the oxidative phosphorylation (OXPHOS) in mitochondria is functional and active [23].

It was later discovered, by means of transmission electron microscopy approaches, that a mitochondrial LDH subunit exists named lactate dehydrogenase B (LDH-B) [1,24]. LDH catalyzes the final, reversible step of the glycolytic pathway, reducing pyruvate to lactate. It is either a homo- or heterotetramer, constituted by subunits called “A” and “B”, whose assembly gives rise to five different isoenzymes [25]. LDH-A (also called the “M” subunit) is mostly present in skeletal muscle and the liver, while LDH-B (also called the “H” subunit) is mainly present in the heart muscle. While the former can be found in cytosol, mitochondria, and organelles, the latter is localized in mitochondria [26].

LDH isoform 5 (LDH-5) is composed of A subunits and features a higher affinity for pyruvate compared to LDH-1, which is composed of B subunits (Figure 1) [27,28]. A sixth isoform, LDH-C4, is present in testis and sperm, in addition to a broad spectrum of human tumors [29]. LDH-5 is overexpressed in tumor cells, and high LDH-A mRNA and protein levels are associated with a significant proportion of BC and poor patient survival [30,31].

It has been shown recently that the LDH-A subunit exerts its pro-oncogenic role through association with RAC1, a GTPase of the RAS superfamily of small GTP-binding proteins [31]. RAC1 is involved in multiple signaling pathways, and its dysregulation leads to cancer development. The levels of LDH-A are directly correlated to the fraction of GTP-bound, active RAC1 in BC cells [31].

LDH-B is a subunit that, as mentioned above, is mainly localized in the mitochondria and produces pyruvate starting from lactate. LDH-B has been shown to be a key driver for non-small cell lung cancer-initiating cells and tumorigenesis [32].

**Figure 2:**
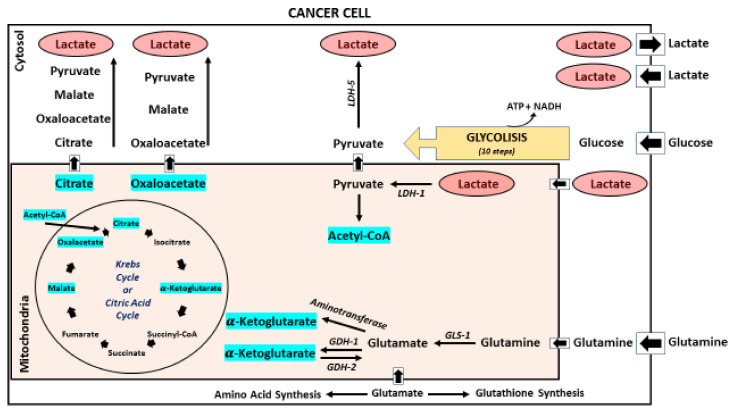
Lactate is produced starting from pyruvate in the cytosol (LDH-5 has the highest affinity for pyruvate). It can be transported through the mitochondrial membrane to serve as a fuel for TCA cycle. Lactate can be converted into pyruvate by the mitochondria-localized isoform (LDH-1). It may also be secreted through the plasma membrane into the extracellular matrix where it contributes to lactic acidosis.

Oxidative metabolism in mitochondria is linked to mitochondria-dependent pyrimidine synthesis. Specifically, the loss of mitochondria OXPHOS can lead to impaired tumorigenesis through the loss of specific nucleotide synthesis rather than mitochondria-dependent ATP production, as recently demonstrated by Bajzikova et al. [32,33]. This evidence is consistent with the discovery that lactate is a primary fuel for tricarboxylic acid (TCA) metabolism, providing the carbon source (mitochondrial pyruvate) and electrons for OXPHOS (Figure 2) [34,35].

LDH-A and LDH-B activities are, at least in part, overlapping, and one subunit can partially substitute for the other. The inhibition of LDH in isolated mitochondria leads to a reduction in mitochondrial metabolism when organelles are cultured in lactate but not pyruvate. This supports the observation that lactate fuels the TCA cycle by being oxidated to pyruvate within tumor cell mitochondria (and not in the cytosol) [24]. The experiments also show that carbon derived from lactate is incorporated into lipids through the conversion to pyruvate, followed by its entrance into the TCA cycle thanks to the pyruvate dehydrogenase complex and citrate synthase [24].

Intriguingly, aerobic glycolysis is a metabolic hallmark of activated DCs [36]. Tumor-infiltrating DCs are critical during tumor immune response and eradication. The anaerobic conditions occurring in the tumor mass are favorable to T helper 1 lymphocyte differentiation [37]. During DC’s activation, the STING pathway plays a crucial role, and its activation leads to increased glycolysis and decreased OXPHOS [36]. Consistently, the knockout of *LDHA/LDHB* in mouse models reduces the numbers, the expression of CD80 and MHC-I, and the antigen cross-presentation ability of tumor-infiltrating DCs. The recent experiments by Hu Z. et al. demonstrate that, eventually, the *LDHA/LDHB* deficiency suppresses DC-mediated antitumor immune responses through aerobic glycolysis impairment. Further support for this hypothesis is that human DCs purified from non-small cell lung cancer (NSCLC) samples feature higher glycolytic rates compared to DCs from paracancerous tissues [36].

*LDHB* expression is associated with poor survival in many human cancers, and the pivotal role of pyruvate–lactate interconversion bears translational implications, as recently demonstrated in glioblastoma (GBM) [27]. Lactate modulates GBM invasion by fueling energy metabolism pathways. This is particularly relevant in brain tumors where the relative pressure of oxygen can be as low as 0.1% in the core area. *LDHA* is upregulated by hypoxia, and lactate production is consistently increased. In these conditions, lactate sustains cell invasion through mitochondrial activity when glucose is absent. Thus, the use of drugs targeting LDH activities represents a promising and innovative approach toward GBM [27].

Lactate has been demonstrated to play a variety of functions in the tumor stroma. Prostate cancer cells display an oxygen consumption rate sustained by the oxidation of the fatty acids stored in lipid droplets (LDs) [38]. Cancer-associated fibroblasts (CAFs) are responsible for the production of lactate that, through ATP-cytrate lyase, guides citrate toward lipid synthesis. For instance, prostate cancer cells exposed to exogenous lactate or to the conditioned medium from CAFs display an increased content of LDs, reflecting an increase in the intracellular lipid deposition [38].

This scenario is consistent with recent evidence showing how BC invasiveness and metastasis are sustained by the increase in fatty acid oxidation in mitochondria and LDs reduction [39,40].

Tumor cells are characterized by a high glucose intake, high energy demand, and a high glycolysis rate. The glycolysis-related molecules involved in this metabolic shift are as follows: glucose transporter 1 (GLUT-1), hexokinase 2 (HK2), pyruvate kinase M2 (PKM2), lactate dehydrogenase (LDH), and lactate transporters (monocarboxylate transporters—MCTs) [41,42,43].

During the Warburg effect, an increasing amount of lactate accumulates in the extracellular environment due to the conversion of pyruvate to lactate through LDH (Figure 2) [1,44]. This affects the different cell types of the TME-like endothelial cells, CAFs, immune cells, and non-cancer stromal cells [44]. The Warburg effect is also fueled by specific changes in the transcriptional program of tumor cells. A notable example is represented by *KRAS*, whose membrane localization depends on glycosphingolipid expression (especially GM3 and SM4) through the aerobic glycolysis system [45]. Glycolysis appears to be pivotal for *KRAS* membrane localization and nanoscale spatial organization. Furthermore, one of the roles of the oncogene *KRAS* is the enhancement of glucose transporters and glycolytic enzymes [45].

Another target affected during the metabolic shift is the 17 KDa membrane-associated protein (*MAP17*), whose expression is hypoxia-dependent and predicts poor prognosis in hepatocellular carcinoma [46]. *MAP17* also activates downstream effectors like AKT and hypoxia-inducible factors 1-alfa (HIF-1α) to enhance the Warburg effect [46]. A further example is represented by the forkhead transcription factor members FOXK1 and FOXK2, which are capable of inducing aerobic glycolysis through the upregulation of the glycolytic enzymatic machinery (HK-2, phosphofructokinase, pyruvate kinase, and LDH) [47].

Membrane glucose transporters *GLUT1* and *GLUT3* are also HIF targets, contributing to the increased glucose uptake during *HIF* activation [1]. Last but not least, *LDH* is also an HIF target gene, and the HIF-1 factors are overexpressed and activated in the hypoxic environment of tumors [48,49].

All the mentioned pathways lead to an increase in lactate production, which is a recognized oncometabolite that has gained considerable attention as an active player in tumorigenesis.

## 3. Lactate in the Major TME Constituents of BC

*Cancer-associated fibroblasts*. The neoplastic parenchimal cells are characterized by intrinsic metabolic requirements and exposed to extrinsic factors like oxygen tension, nutrient availability, and pH (these latter are determined by the TME). A pivotal component of the TME is represented by stromal fibroblasts, commonly referred to as CAFs [44]. CAFs contribute to the architecture and functions of the stroma by releasing cytokines, signaling factors, and depositing extracellular matrix [50]. Features of the TME affect the tumor at various levels, leading to an inefficient tumor vasculature that inefficiently delivers nutrients and removes catabolic products, including lactate [44,51]. In prostate cancer TME, an interplay between CAFs and prostate cancer cells has been demonstrated. In this model, CAFs grown in the presence of cancer cells undergo a series of changes, including an increase in GLUT-1 and MCT4 expression, leading to an increase in the glucose intake and lactate release. Thus, prostate cancer cells metabolize the CAF-derived lactate [52]. CAFs have been recently described as molecular biomarkers in BC. They are specialized, activated fibroblasts with high metabolic plasticity that support tumor growth through the secretion of cytokines, growth factors, differentiation factors, and extracellular matrix remodeling molecules [53,54,55]. It has been recently demonstrated that CAFs fuel BC cells by lactate transfer [56]. Specifically, hypoxia drives the glycolytic activity of CAFs through ATM oxidation, GLUT1 phosphorylation, and PKM2 overexpression. Lactate produced by CAFs eventually drives BC cell invasion through the activation of the TGFβ1/p38 MAPK/MMP2/9 signaling axis and fueling mitochondrial OXPHOS [56]. Overall, this hypoxia-derived lactate leads to an acceleration of cancer cell invasion and an increase in the in vivo metastatic potential [56]. Most recently, a new subpopulation of CAFs generated by lactic acidosis has been described, consisting of the acquisition of a CAF-like phenotype by adipocyte precursors. These adipocyte-derived CAFs display protumorigenic activity, sustaining proliferation, migration, invasion, and therapy resistance to BC cells [57].

*Immune cells.* A consequence of inefficient perfusion is tissue hypoxia, which is a cause of TME acidification. This acidification also affects the immune response toward tumor cells in terms of competition for nutrients and the capability to switch between metabolic pathways [58]. Activated T cells switch from oxidative to glycolytic metabolism even in the presence of oxygen during the effector response. This is aimed at biosynthetic pathways that are functional for effector functions and proliferation [58]. Activated T cells are considered predominantly glycolytic and characterized by an increased uptake of anabolic precursors (glucose and amino acids) and the production of lactate. Specifically, effector T cells require a high influx of glucose and glutamine. After proliferation and pathogen clearance, effector T cells undergo a metabolic shift toward OXPHOS metabolism. This reprogramming is necessary for the switch from effector to memory cells, the latter being characterized by mitochondrial respiration and OXPHOS metabolism [59]. It is well known that hypoxic and acidic TME hamper anti-tumor immune responses while favoring T-regulatory cells and tumor-associated macrophages. Within the TME, there is nutrient and metabolic competition between immune and cancer cells [60]. Cancer cells adopt to counteract anti-tumor immune response by depleting some essential nutrients. This is the case of glucose, for instance, which is used by rapidly proliferating cells like tumor-infiltrating T lymphocytes to support proliferation and differentiation [61]. As already mentioned, the high glucose consumption characteristic of tumor cells leads to an overproduction of lactate, which is exported outside of the cells by means of the MCTs (especially MCT4). The consequent acidification of the TME represents an immunosuppressive factor and promotes tumorigenesis through IL-17- and IL-23-mediated inflammation [62]. Recently, a lactate score demonstrated its potential as an independent prognostic factor in BC. This score encompassed 12 lactate metabolism-related pathways that were screened and found to be enriched in BC. The analysis led to a panel of lactate-related genes (LRGs) [63]. The BC samples, classified according to the expression levels of the selected LRGs, can be classified on the basis of a new lactate score. The lactate score can evaluate the TME immune cell infiltration and the prognosis of BC patients. Thus, patients with high or low lactate scores also showed different responses to immunotherapies, clinicopathological features, and anti-PD-1 drug susceptibilities. Specifically, a low lactate score was associated with immune activation with increased CD8^+^ T infiltration and inflamed TME [63].

*Adipose tissue*. Mammary adipocytes represent a central constituent of BC TME. Additionally, defined as “cancer-associated adipocytets” (CAAs), they represent up to 90% of breast tissue in BC and emerged as pivotal players in communication with cancer tissue through lactate [64]. CAAs, in contrast to mature adipocytes, feature a decreased content in lipid droplets accompanied by a mobilization of free fatty acids and high-energy metabolites [65]. Lipid reprogramming involving CAAs is known to mediate several malignant processes like progression, metastasis, and therapy resistance. Therefore, it gained considerable attention as a research field for therapeutic purposes [66]. Lactate, as an energetic fuel and a redox-maintaining molecule, is particularly interesting in BC since this tumor is highly dependent on the microenvironment and its metabolism [67]. BC is characterized by the fact that tumor cells are embedded in adipose tissue. CAAs communicate with tumor cells in a paracrine way through the secretion of growth factors, adipokines, and proinflammatory cytokines and, being at the crossroads of glucose and lipid metabolism, refurnish cancer cells with nutrients like fatty acids, ketone bodies, and glycerol [64,68]. Lactate production by adipocytes can occur independently of glucose availability, both when glucose is available and unavailable, demonstrating a novel function for adipocytes as lactate producers [69]. Lactate functions as a metabolic fuel (thanks to the mitochondrial conversion into pyruvate) and also as a messenger between CAAs and cancer cells. This link becomes particularly important in obese women, where the dysfunction of resistant adipocytes affects the communication between CAAs and cancer cells, eventually promoting cancer aggressiveness [64]. It is notable that BC cells are known to induce extensive lypolysis of CAAs and induce them into myofibroblasts, thus supporting cancer cell growth [68]. The most recent experimental evidence consistently demonstrates that mammary adipocytes, during BC progression, de-differentiate into myofibroblasts and macrophage-like cells through metabolic reprogramming. Lactic acidosis promotes the adipocyte–myobroblast transition in a pre-fibrotic and pro-inflammatory environment [57,68]. These myofibroblasts have lost the adipocyte differentiation markers, produce pro-inflammatory cytokines, and are responsible for extracellular matrix synthesis/deposition and remodeling, eventually exerting a protumorigenic role [57].

## 4. Lactate in BC Metabolism and Tumorigenesis

Cancer is now the second-leading cause of death worldwide after cardiovascular disease [70]. BC is the most commonly diagnosed cancer type in women (only 0.5–1.0% occur in men), accounting for one in eight diagnoses worldwide, and in 2020, there were about 2.3 million women diagnosed with BC globally and about 685,000 deaths from this disease. Among the risk factors there are age, obesity, certain mutations, and family history of BC [71]. The treatment options are represented by surgical removal, radiation therapy, chemotherapy, and hormonal or targeted therapies [71].

As introduced above, TNBC is defined by the simultaneous absence of ER, PR, and HER2 amplification and is associated with a poor prognosis. The molecular characterization of BC led to the identification of four “intrinsic subtypes” based on the gene expression patterns [72]. The basal-like subtype (that shows positivity for basal and myoepithelial markers) overlaps with TNBCs since it lacks hormone receptors and HER2 amplification. The two subtypes share the same immunophenotype and represent the most aggressive and incurable BC variants, characterized by the highest metastatic potential [72]. The absence of the specific relevant receptors implies that TNBCs are no longer treatable with hormone- or anti-HER2-targeted therapy. The evidence supports the role of the Warburg effect in the proliferation, metastasis, recurrence, drug resistance, and immune escape of TNBCs [73].

Also, pre-malignant lesions develop in a TME characterized by hypoxia. Ductal carcinoma in situ (DCIS) is considered the earliest form of BC [74]. DCIS develops in poor metabolic conditions since hyperplasia forces the cells toward the ductal lumen and away from the blood vessels, exceeding the oxygen diffusion capability. As a consequence, they display a glucose metabolism skewed toward fermentation, even in normoxia [74]. The consequent production of lactic acid determines the acidic environment of periluminal areas, which can be quantitatively measured (by membrane-associated Lamp2b). Thus, the interplay among nutrient deprivation, hypoxia, and acidity contributes to the selection of a cancer cell phenotype that is adapted to survive [74].

Obesity has been linked to a higher risk of BC, advanced disease at diagnosis, and poor prognosis, especially in postmenopausal women [75]. Estrogen receptor positive (ER^+^) and ER negative (ER^−^) BC display biological differences, entailing different treatments, prognoses, and patterns of risk factors [76]. Despite the emphasis on estrogen activities, which in postmenopausal are principally derived from the adipose tissues, other factors may prove to be equally important.

**Figure 3:**
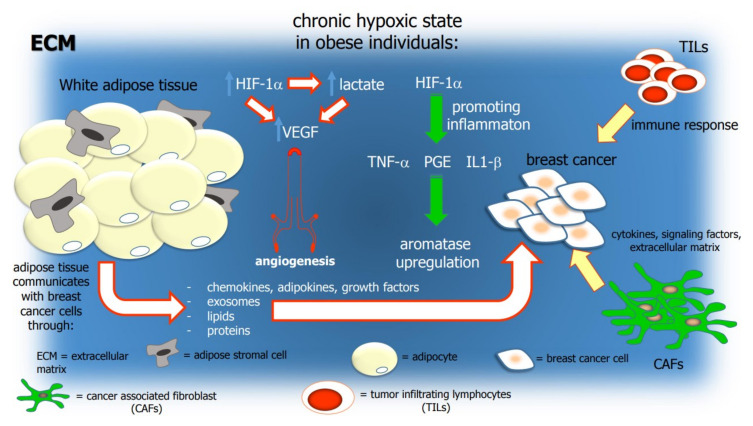
Schematic representation of the interactions of the main constituent of TME with breast cancer cells. The hypoxic environment of breast cancer triggers the overexpression of HIF-1α, which fuels lactate production. These two lead to the overexpression of VEGF, promoting angiogenesis and tumor invasiveness. HIF-1α also leads to proinflammatory molecule secretion (like TNF-α, PGE, and IL1β) that sustains aromatase overexpression.

In obese individuals, adipose tissue is characterized by a chronic state of hypoxia, leading to the increased expression of HIF-1α. This is a well-known inflammatory cascade promoter stimulating the tumor necrosis factor alfa (TNF-α) and prostaglandin E2 (PGE2) activity in the overexpression of aromatase in the stromal surrounding adipose tissue, particularly that of the breast (Figure 3) [77].

HIF-1α regulates many pivotal pathways in normal as well as cancerous cells, including angiogenesis, cell proliferation, survival, and tumor progression, through the regulation of growth promoters, oncogenes, glycolytic pathways, and pH [78,79]. Hypoxic conditions impose tumor cells to reprogram their metabolism. The hypoxia-induced change could be the first step in promoting the tumor cell metabolism with oncometabolites release. The switch to aerobic glycolysis is a central feature of solid tumor microenvironments, fueling rapid growth with elevated glucose consumption [80,81]. Malignant transformation is underscored by a genetic mutation activating *HIF-1α* and the subsequent upregulation of *LDH-A*, leading to the increased conversion of pyruvate to lactate [79,80]. The accumulation of lactate generates an acidic environment, suppressing normal immunological functions by T and NK cells while promoting an accelerated local invasion [82].

BC is directly influenced by metabolism. Obese and type 2 diabetes patients have larger tumors at diagnosis and a worse prognosis, associated with higher risks of metastatic disease [83]. Adipose tissue communicates with cancer cells in the breast, contributing to cancer progression through the release of signaling molecules, extracellular matrix deposition, and energy metabolites (Figure 3) [83].

Recently, the role of lactate-regulating gene expression has been demonstrated in the ER^+^ MCF7 cell line. In MCF7 cells, lactate causes significant increases in the expression of genes involved in cell signaling, cell growth, angiogenesis, and proliferation. Consistently, the vascular endothelial growth factor (VEGF) is produced by tumor cells in response to stimuli such as hypoxia and lactate [84]. It is well known that the activation of the VEGF signaling pathway is associated with clinical outcomes in BC patients and represents a therapeutic target through the use of Bevacizumab [85].

Furthermore, chaperone-mediated autophagy promotes BC angiogenesis through the regulation of HK2-dependent aerobic glycolysis [86].

Finally, the protumorigenic contribution of lactic acidosis influences the adipocyte precursor cells in the TME. These may develop into a subpopulation (cancer-associated adipocytes—CAAs) that sustains the proliferation, migration, invasion, and therapy resistance of BC cells in vitro [57].

In light of this evidence, the recent development of glycolysis inhibitors represents a desirable therapeutic opportunity. Here, we report just some of the many molecules targeted to specifically inhibit this metabolic pathway.

Galloflavin, an LDH inhibitor, induces tumor regression in vitro in human BC cells with different glycolytic attitudes by inhibiting the bioenergetic metabolism of cancer cells and underpinning the role of lactate as a response marker [87].

More recently, the technology of proteolysis-targeting chimera has been applied to discover new compounds that are capable of degrading HK2, the rate-limiting enzyme of glycolysis [88]. Following glycolysis inhibition, the HK2-degraders induce mitochondrial damage and pyroptosis, eventually leading to immunogenic cell death. This last effect reactivates the antitumor immune response, demonstrating how HK2 inhibition may also reverse an immunosuppressive TME [88].

The combination therapy of the glycolysis inhibitor 2-deoxyglucose with the anti-diabetic drug Metformin showed promising results on TNBCs, thanks to the high dependence on glucose metabolism [89]. Also, the drug Pimozide, approved as an antipsychotic, displays antitumor activity in a variety of cancer cells, including breast. It exerts pleiotropic activity by upregulating p53, downregulating the expression of pyruvate kinase M2, and eventually inhibiting the Warburg effect [90].

As described above, lactate should not be considered a mere end-product of tumor metabolism. In fact, its role in cancer biology as a substrate to actively fuel tumor and TME cells is well known [91]. This has also been widely demonstrated in BC, both in the animal model and in human-derived tissues. TNBC cells, for example, can switch to lactate as a primary source of energy under conditions of glucose depletion [92]. Lactate, probably through the conversion into pyruvate, directly sustains the TCA cycle, assuming a pivotal role in NADPH production and bioenergetic requirement fulfillment in the glucose-deprived TME [93]. Key factors in the utilization of lactate as cellular fuel consist in overexpression/increased activity (achieved by post-translational modification) of LDHB, thatmainly oxidizes lactate into pyruvate [94], and MCT1, which is mainly associated with lactate cellular imports, whose overexpression by cancer cells in subtypes of BC was found to be associated with poor prognosis [95,96]. The biochemical modifications underlie the establishment of an efficient crosstalk between cancerous and non-cancerous cells in TME, in which glycolytic cells from hypoxic regions of the tumor and CAFs feed cancer cells (residing in more oxygenated regions) with lactate derived from glycolysis [97].

Different BC subtypes undergo specific oncogenic alterations in cellular pathways and TME interactions [98]. For example, high MYC gene expression was found to be more frequent in the TNBC subtype than in ER^+^ and HER2^+^ tumors, and it was strictly associated with genes regulating glucose metabolism in ER^−^ but not in ER^+^ cases, suggesting a differential role of this key oncogene in driving glucose metabolism in different BC subtypes [99]. The effects of this and other pathways ultimately result in differences in metabolic reprogramming and, hence, diverse metabolic phenotypes can be observed among BC subtypes [100]. In fact, metabolomic studies revealed that glycolytic intermediates and lactate tumor concentrations differ depending on the BC subtype, with higher levels of the latter being observed in TNBC and HER2^+^ cases compared to ER^+^ tumors [101].

Among the biochemical and molecular factors contributing to metabolic differences in BC subtypes, the expression of ER makes BC cells susceptible to estrogen-mediated effects on glucose and lactate metabolism [102]. In fact, the existence of a link between estrogen and lactate metabolism in BC cells has been well known for decades [103]. BC cells treated in vitro with estrogen show increased glucose utilization and lactate production, with the addition of estradiol being able to induce over-expression of the enzyme LDH [104,105]. LDH upregulation can be, at least in part, due to the estrogen-mediated regulation of several micro RNA genes controlling glycolytic metabolism [106]. Accordingly, tamoxifen, an anti-estrogen therapy used to treat ER^+^ BC patients, reduces the rate of glycolysis and lactate production in sensitive BC cells [107].

Not surprisingly, metabolic adaptations and the bioenergetic crosstalk existing between BC cells and their tumor microenvironment seem to play an important role in the development of anti-estrogen therapy resistance. In fact, tamoxifen-resistant BC cells display altered cellular pathways linked to aerobic glycolysis compared to parental BC cells [108]. Moreover, resistance to tamoxifen is strictly linked to the presence of metabolically aberrant CAFs in the TME. These show increased glycolysis and secretion of lactate that is up taken and metabolized by oxidative cancer cells, overcoming the metabolic effects resulting from a blockade of the estrogen stimulation [109]. Overall, all these metabolic cellular adaptations suggest possible alternative therapeutic strategies of clinical relevance. For instance, the simultaneous inhibition of LDHA, an enzyme found to be overexpressed in tamoxifen-resistant cells, was capable of inducing re-sensitization to the anti-estrogenic drug [110]. Again, co-administration with a glycolysis inhibitor or suppression of the protoncogene c-Myc (which is overexpressed in tamoxifen-resistant cells compared to parental cells) was able to reduce or abolish anti-estrogen resistance [111].

## 5. Circulating Lactate in Breast Cancer

As described above, the biochemical phenotype characterized by the upregulation of glycolytic enzymes such as LDH-A and increased lactate production is an essential component of the metabolic reprogramming of tumor cells for the majority of human malignancies and BC in particular, involving the contribution at the cellular level from clinically relevant oncogenic molecular lesions such as HER2 [112,113,114].

From a clinical standpoint, elevated levels of the circulating enzyme LDH have been historically used in oncology as a marker of poor prognosis, which is usually attributed to an elevated tumor burden and cancer metabolism [25]. This is true even for BC patients, for whom increased circulating levels of LDH have been associated with reduced survival [115]. Regarding the possible role of circulating lactate levels in the clinical management of BC patients, however, the available evidence is scarce and far from definitive.

In normal conditions, circulating lactate derives from the anaerobic metabolism of a wide variety of organs and tissues, with the skeletal muscle being the major contributor. From plasma, lactate is primarily metabolized back into glucose through gluconeogenesis, mainly by the liver and, to a lesser extent, by the kidneys [116].

Clinical conditions characterized by severely increased (≥4–5 mmol/L) plasma lactate concentrations are usually accompanied by acidemia (i.e., lactic acidosis) and are classified into two main categories. Type A lactic acidosis is secondary to tissue hypoxia or hypoperfusion, whereas type B arises during impaired oxygen availability or delivery, although nonhypoxic mechanisms are probably relevant in inducing increased lactate concentrations even in the former [117]. Examples of type A lactic acidosis are circulatory shock, sepsis, burns, and regional tissue ischemia; type B includes severe liver failure, uncontrolled diabetes mellitus, hereditary mitochondrial defects, thiamine deficiency, and drugs or toxins interfering, directly or indirectly, with lactate metabolism [118].

The tumor-associated Warburg effect is a well-known cause of type B lactic acidosis [119]. Nonetheless, lactic acidosis in oncological patients is generally considered of multifactorial origin [120].

The evidence regarding increased circulating lactate levels in oncological patients is mainly derived from published case reports [121]. In particular, eleven cases from ten papers reporting lactic acidosis in BC patients were retrieved from our search (Table 2) [122,123,124,125,126,127,128,129,130,131].

The presence of liver metastasis with associated liver dysfunction has been suggested to be a relevant factor contributing to increased concentrations of circulating lactate in the oncological patient, given the central role of the abdominal organ in lactate metabolism [132]. This was suggested by the high prevalence of liver involvement in cases of lactic acidosis developing in oncological patients, especially in solid tumors [133]. For example, out of the eleven cases of BC complicated with lactic acidosis, definite liver involvement was reported in at least eight of them [122,124,126,127,128,129,130,131]. However, it must be stated that the exact role of liver metastasis/dysfunction in the development of lactic acidosis in oncological patients is far from completely understood, since a significant number of cases arise without overt liver involvement [121]. Moreover, lactic acidosis is not a common complication of liver failure alone [134].

As stated above, drugs are a possible cause of type B lactic acidosis. Antineoplastic agents are among the pharmacological principles implicated in derailing lactate metabolism [135]. Oncological patients are usually exposed to a multitude of pharmacological therapies and, hence, are at increased risk of drug–drug interactions [136]. For example, in the case reported by Lagampan C et al., lactic acidosis in a BC patient was ascribed to an accumulation of the anti-diabetic drug metformin in the setting of an acute kidney injury due to the inhibitory effect of the antineoplastic Ribociclib on its renal elimination and catabolism [123].

Among other possible contributing causes for hyperlactacidemia and lactic acidosis in BC patients, two cases of pulmonary tumor microembolism induced a rapidly fatal state of cardiopulmonary failure with hypoxia/hypoperfusion and associated lactic acidosis [125]. Infection and sepsis also remain an important cause of morbidity and mortality in oncological patients and possible contributors for increased lactate levels [137]. For example, in the case described by Al Qahtani et al., a significant increase in circulating lactate followed the development of spontaneous bacterial peritonitis, complicating severe liver involvement by BC with hepatic dysfunction and the formation of ascites [124]. Finally, it is worth mentioning thiamine deficiency as a possible cause of hyperlactacidemia in oncological patients. Thiamine (vitamin B1) is a cofactor for pyruvate dehydrogenase; its deficit impairs the conversion of pyruvate to acetyl CoA for the citric acid cycle, instead inducing an increased conversion to lactate [138]. While it has been reported as a possible contributing cause In only one BC patient [122], thiamine deficiency could develop in oncological patients as a complication of long-term total parenteral nutrition with inadequate supplementation [139].

Nonetheless, tumor-associated Warburg effects appear to be a predominant cause of hyperlactacidemia and lactic acidosis in a significant percentage of tumor cases [112]. Indirect evidence for this is the observation that anti-tumor therapy has been reported to be the sole intervention capable of inducing a significant reduction in lactate levels in these patients [120]. On the other hand, alkalinizing therapy with intravenous or oral sodium bicarbonate has proven to be ineffective in mitigating or resolving this metabolic derailment [140]. This was clearly exemplified by the BC patient described by Brivet et al., in which the hyperlactacidemia progressively worsened in spite of a bicarbonate infusion [128].

Regardless of the underlying cause(s) and therapies, lactic acidosis is generally considered a complication of an advanced oncological disease and has been associated with very poor outcomes in oncological patients, based on the published reports [121]. Out of the eleven BC patients reported, eight had a survival time of only a few hours/weeks after the onset of lactic acidosis [122,124,125,127,128,129,131].

Apart from the aforementioned cases with overt lactic acidosis, the evidence linking circulating lactate levels and clinical outcomes in BC patients is still lacking. Of note, Lende et al. conducted a randomized controlled trial reporting that, in a subgroup of estrogen receptor positive cases, operable BC patients who received oral pre-operative carbohydrates had higher tumor proliferation (determined as the mitotic activity index in the surgical pathology specimen) and poorer relapse-free survival rates in respect to fasting patients [141]. Among the speculated mechanisms explaining these findings, the authors suggested a proliferative/survival benefit for BC cells induced by carbohydrates and mediated directly via the Warburg effect or indirectly via the insulin axis. This was later confirmed with a metabolomics methodology that detected significant differences in the metabolic profile of serum and tumor tissues of these patients compatible with these assumptions, including an increase in serum lactate in the carbohydrate-treated group that is not usually observed in healthy or diabetic individuals after a carbohydrate load [142].

Other studies have proven that the glucose metabolism of BC cells is probably influenced by nutrient and substrate availability and by nutrition. For example, plasma lactate concentrations were significantly lower after 60 days of fish oil supplementation in BC patients during adjuvant chemotherapy in respect to a control group [143]. Moreover, another study found that 12 weeks of a ketogenic, high-fat, and very low-carbohydrate diet were associated with a significant reduction in circulating lactate levels in comparison to a control group [144].

In conclusion, the evidence regarding circulating lactate and BC is still scarce and mainly related to a few cases reporting lactic acidosis, along with some studies that evaluated the metabolic effects of nutrient/substrate availability in BC patients. These studies suggest a multifactorial origin for the increased circulating lactate levels seen in BC patients and a poor outcome associated with hyperlactacidemia and lactic acidosis. However, more studies are definitely needed to clarify the link between circulating lactate and clinical outcomes in BC patients.

## 6. Concluding Remarks

The scientific evidence published during the last few years in authoritative journals converges on the central role of lactate as an active metabolite. Lactate is no longer just a glycolysis byproduct but a fuel for the TCA cycle, OXPHOS, and a protumorigenic molecule. Lactate in the TME of BC promotes tumorigenesis through mechanisms triggered by lactic acidosis. These encompass the production of VEGF, the pivotal role of CAFs, and the interplay between white adipose tissue and BC cells. Lactate also represents a carbon source for lipid synthesis and promotes LD formation. LDH is also a target gene for HIF-1α in a TME frequently characterized by hypoxia. Furthermore, the limited amount of evidence available thus far on circulating lactate in BC patients reviewed in the present manuscript points toward a positive correlation between lactate levels and a worse prognosis. Collectively, this oncometabolite deserves attention during disease monitoring and bears great potential as a biomarker in BC.

## Figures and Tables

**Table 1:** Milestones in lactate metabolism and cancer.

Year of Publication	Article	Description
1923	[9]	Increased production of lactate and acidification by tumor cells upon addition of glucose, independent of oxygen.
1972	[10]	Aerobic glycolysis in tumors named “Warburg effect”.
1997	[11]	Transcription factor Myc transactivates the gene encoding the enzyme LDHA, which links oncogenic tumor pathways and metabolic rewiring that takes place during carcinogenesis.
2002	[12]	Lactate, mainly through conversion into pyruvate, stimulates HIF-1 accumulation through increased protein stability and gene expression; lactate acts as a signaling molecule.
2008	[13]	Lactate produced in hypoxic regions is taken up by oxygenated cancer cells through “metabolic symbiosis” between cancer cells and tumor microenvironment.
2011	[14]	Rewiring of energy metabolism elevated to the status of emerging hallmark of cancer.

**Table 2:** Case reports of lactic acidosis in breast cancer patients.

Year of Publication	Authors	Age	Liver Metastasis	Lactate Peak (mmol/L)	Therapy	Survival after the Onset of Lactic Acidosis	Notes
1980	[130]	61	Yes	17.2	Chemotherapy and oral bicarbonate	Alive after 2 weeks	Self-discharge against medical advice.
1983	[129]	36	Yes	13	Chemotherapy and bicarbonate	6 days	
1984	[128]	54	Yes	27	Chemotherapy (initiated 3 months prior for stage IV breast cancer); bicarbonate infusion	2 days	Lactate increased despite bicarbonate infusion.
1985	[131]	67	Yes	16.6	Chemotherapy	10 days	
1992	[126]	36	Yes	5	Bicarbonate and chemotherapy/gonadotropin antagonist (6 cycles)	Alive after 14 months	Onset at 36 weeks of pregnancy.
1992	[127]	67	Yes	13	Chemotherapy and bicarbonate	8 days	
2006	[125]	29	NR	12	Only support therapy	Hours	Tumor emboli in lungs.
2006	[125]	46	NR	12	Only support therapy	Hours	Tumor emboli in lungs; mild lactic acidosis three months before emolization not resolved after stopping antiretroviral therapy.
2011	[122]	86	Yes	7.5	Thiamine, i.v. bicarbonate, chemotherapy	Few weeks	Mild thiamine deficit; no reduction in lactate levels after supplementation.
2019	[124]	26	Yes	16.9	Support/antibiotic therapy	14 days	Presentation with sign/symptoms of liver failure; spontaneous bacterial peritonitis; death of liver failure and coagulopathy.
2021	[123]	62	0	13.7	Ribociclib	Alive after 12 months	Interaction between Ribociclib and Metformin in the setting of acute kidney injury likely causing lactic acidosis; recovery after Ribociclib discontinuation and renal replacement therapy.

## Data Availability

Any further information is available upon request from the authors.

## Abbreviations

ATP = adenosine triphosphate; BC = breast cancer; CAAs = cancer-associated adipocytes; CAFs = cancer-associated fibroblasts; CD80 = cluster of differentiation 80; DCIS = ductal carcinoma in situ; DCs = dendritic cells; ER = estrogen receptor; FOXK1 = forkhead box protein K1; FOXK2 = forkhead box protein K2; GBM = glioblastoma; GLUT-1 = glucose transporter-1; GM3 = monosialodihexosylganglioside-3; GPT = guanosine triphosphate; HER2 = human epidermal growth factor-2; HK2 = hexokinase-2; KRAS = Kirsten rat sarcoma viral proto-oncogene; LDH = lactate dehydrogenase; LDs = lipid droplets; MAP17 = membrane-associated protein-17; MCTs = monocarboxylate transporters; MHC-I = major histocompatibility complex class I; MYC = myelocytomatosis oncogene; NAD^+^ = nicotinamide adenine dinucleotide; NK = natural killer; NSCLC = non-small cell lung cancer; OXPHOS = oxidative phosphorylation; PGE2 = prostaglandin E2; PKM2 = pyruvate kinase M2; PR = progesterone receptor; RAC1 = ras-related C3 botulinum toxin substrate 1; RAS = rat sarcoma virus; SM =sphyngomielin; STING = stimulator of interferon genes; TCA = tricarboxylic acid; TME = tumor microenvironment; TNBC = triple-negative breast cancer; TNF-α = tumor necrosis factor alfa; VEGF = vascular endothelial growth factor; AKT = AKT serine/threonine kinase 1.
